# Vulnerability of sea turtles and fishes in response to two catastrophic Caribbean hurricanes, Irma and Maria

**DOI:** 10.1038/s41598-019-50523-3

**Published:** 2019-10-03

**Authors:** J. K. Matley, S. Eanes, R. S. Nemeth, P. D. Jobsis

**Affiliations:** 1 Present Address: Great Lakes Institute for Environmental Research, University of Windsor, Windsor, Ontario Canada; 2 Center for Marine and Environmental Studies, University of the Virgin Islands, St. Thomas, VI USA

**Keywords:** Behavioural ecology, Environmental impact

## Abstract

Extreme weather events (e.g., cyclones, floods, droughts) are capable of changing ecosystems and altering how animals obtain resources. Understanding the behavioural responses of animals being impacted by these natural events can help initiate and ameliorate conservation or management programs. This study investigated short- and long-term space-use of the critically endangered hawksbill sea turtle (*Eretmochelys imbricata*), as well as five species of fishes and stingrays, in response to two of the most destructive Caribbean hurricanes in known history – Irma and Maria, which were at their peak intensity when they passed the US Virgin Islands in September of 2017. Using passive acoustic telemetry in St. Thomas, US Virgin Islands, we show a variety of short-term behavioural patterns initiated across species to reduce exposure to the strong environmental conditions, such as moving to deeper habitats within the study area. Although short-term expansion of activity space was evident for several sea turtles, long-term impacts on space-use and body condition were limited. In contrast, southern stingrays (*Hypanus americanus*) left the study area shortly after the hurricanes, suggesting vulnerability stemming from altered habitat, prey availability, or temperature/oxygen profiles. This study shows the strong spatial resilience of several nearshore species despite exposure to two consecutive category 5 hurricanes.

## Introduction

Extreme weather events have the capacity to severely damage or disrupt ecosystems across a gradient of spatial and temporal scales^1^. Immediate impacts may cause mortality or displacement, and readily affect an animal’s access to fixed resources such as food and shelter^2,3^. Similarly, physical alteration of habitat (e.g., temperature, nest sites, visibility, migration routes, etc.) can lead to suboptimal physiological conditions^4,5^. If deleterious conditions persist or respite from the disturbance is not attained, impacts may become more critical affecting survival through metabolic/nutritional deficiencies, disease, or phenotypic/genotypic selective pressures, among others^6–8^. Overall, these consequences can alter population or community dynamics causing multiplicative effects throughout the ecosystem^9^. For example, the co-existence of certain species and differences in species diversity are strongly driven by mechanistic drivers relating to moderate or patchy disturbances (intermediate disturbance hypothesis^10,11^). For highly mobile species, emigration or escape from the disturbed area, either before, during, or after, may increase chances of survival^12,13^, whereas individuals with limited escape ability may have to rely on other adaptations^14,15^. Resilience to environmental disturbance is a key ecological concept driving the survival of individuals/species as these events have increasingly become more intense, especially in the Western Atlantic Ocean and Caribbean^16^. Indeed, natural and human-induced disturbances augmented by climate change are affecting organisms in aquatic and terrestrial ecosystems worldwide at an alarming rate^17–19^. Species that lack specific adaptations to escape or endure drastic changes in environmental conditions, as well as those with specialized resource needs or that face extinction/extirpation are particularly at risk posing specific conservation concerns^20,21^. Further, predicting how animals will respond to or are affected by extreme weather provides relevant context for disaster planning and the ecological impacts that ensue. This is especially applicable for management in areas that rely directly on access to natural resources for social or economic subsistence^22,23^ (e.g., tourism, fishing, and agriculture).

**Figure 1:**
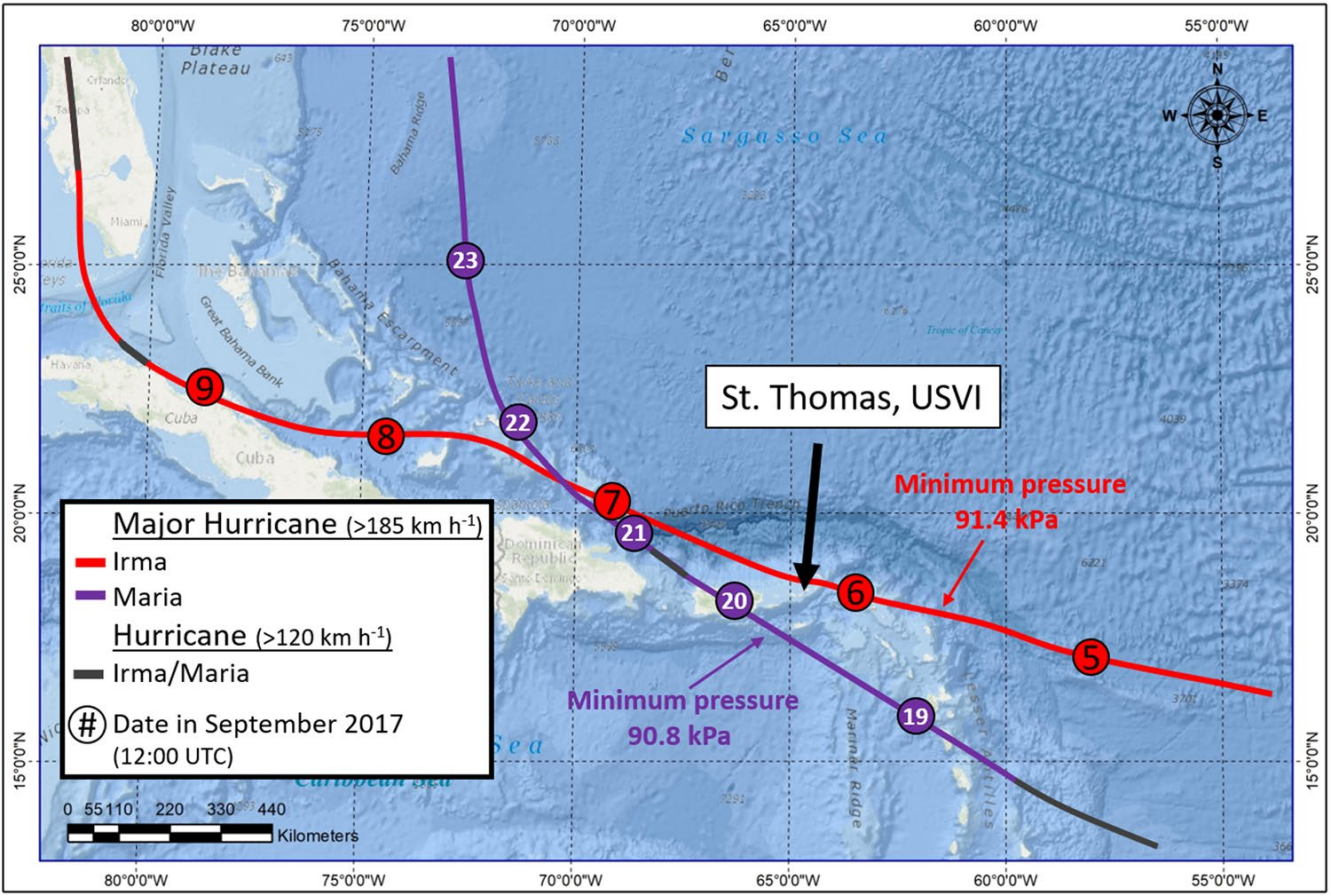
Approximate track of Hurricanes Irma and Maria during September 2017 including the location of St. Thomas, US Virgin Islands. This map (with self-created symbols/shapes/text) was created using ESRI (Environmental Systems Resource Institute; http://www.esri.com/software/arcgis) ArcMap software, version 10.6. Basemap sources include: Esri, GEBCO, NOAA, Garmin, HERE, and other contributors.

In September 2017, two category 5 hurricanes – Irma and Maria – made destructive landfall across the eastern Caribbean (Fig. 1). At its peak, Hurricane Irma produced winds as strong as 155 kts (~287 km h^−1^)^24^ and has been regarded as one of the most powerful storms in the Caribbean in known history. The hurricanes hit St. Thomas, US Virgin Islands (18.33°N, 64.97°W) – a small island (~50 km^2^) and popular vacation destination – on September 6^th^ (Irma) and September 20^th^ (Maria) causing widespread damage. Hurricane Irma was at its peak strength (atmospheric pressure: ~91.5 kPa) when the center of the eye passed 30 km north of St. Thomas with the southern eye-wall passing along the island’s northern coast. Hurricane Maria (~91.3 kPa, 259 km h^−1^) was also near its peak when it passed 70 km south of St. Thomas^25^.

**Figure 2:**
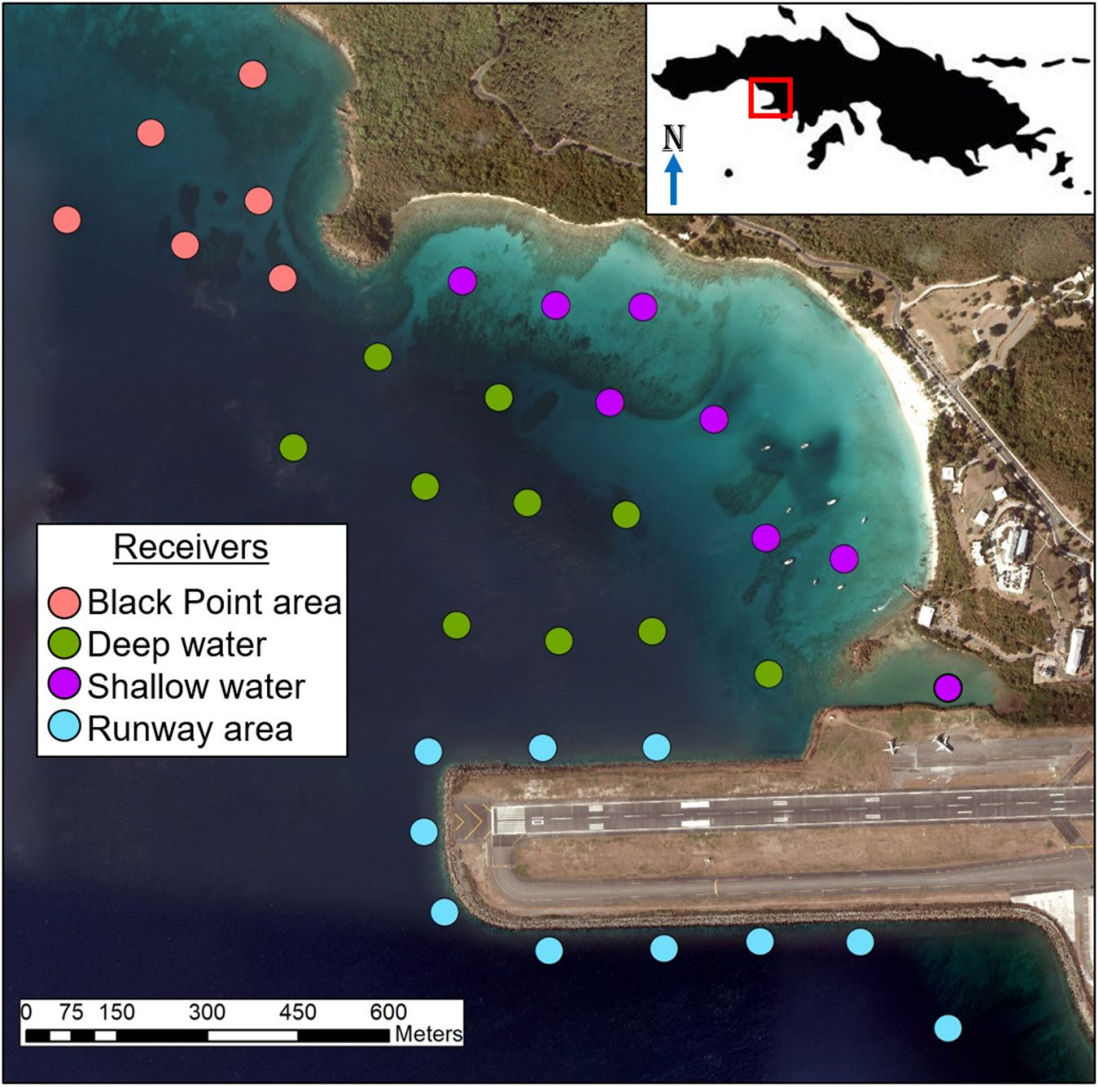
Brewers Bay receiver array (18.3425°N, 64.9800°W) located in the US Virgin Islands (see map inset) with four habitat categories identified. Only receivers that were present prior to and after Hurricane Irma are included (34 receivers). The habitat categories were broadly categorized into four groups: shallow (<10 m), deep (>10 m), Black Point (the northern point within Brewers Bay), and runway (the southern part of Brewers Bay). This map (with self-created symbols/shapes/text) was created using ESRI (Environmental Systems Resource Institute; http://www.esri.com/software/arcgis) ArcMap software, version 10.6. Basemap sources include: SIO, NOAA, US Navy, NGA, GEBCO, and Google Earth.

Due to the unpredictable and destructive nature of hurricanes/cyclones, few studies exist tracking the movements of marine species at extensive temporal scales relevant to these events. Additionally, most reports lack the spatial resolution needed to track marine animals consistently before and after disturbances. In this paper we describe the results of an acoustic telemetry study, located in Brewers Bay, St. Thomas (Fig. 2), that provided a unique opportunity to analyze the movements of a diversity of marine species before, during, and after these hurricanes, offering unique insight on the behavioural responses to extreme weather events. We investigated the spatial patterns of juvenile sea turtles, stingrays, and tarpon, as well as three species of snappers across varying temporal scales in response to two consecutive extreme environmental events – Hurricanes Irma and Maria. Emphasis was placed on comparing regular weekly spatial patterns of individuals across detection periods relative to behavior immediately following Irma – the more severe and destructive hurricane. Several spatial metrics were used to identify the size and locations of space used throughout the period individuals were tracked. We predicted that the short-term (≤week) impacts of Hurricane Irma would cause shifts in regular spatial patterns, particularly in shallower waters where habitat disturbance would presumably be greatest. Long-term impacts were expected to be minimal except for those individuals that were driven outside Brewers Bay due to the hurricanes to find more suitable habitat.

## Results

### Hawksbill turtles

**Figure 3:**
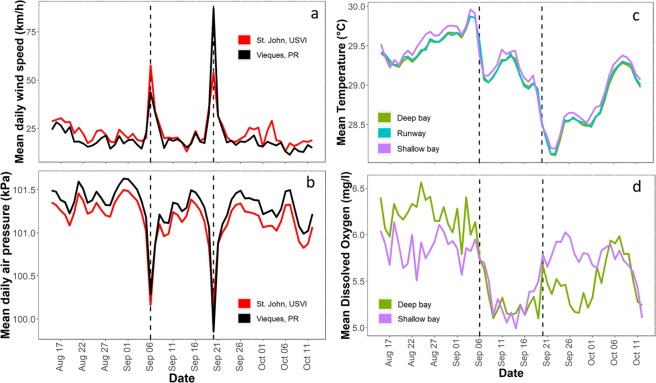
Mean daily values of environmental parameters including wind speed (**a**) and air pressure (**b**) taken from weather stations in St. John, USVI and Vieques, Puerto Rico, as well as water temperature (**c**; n = 12 loggers) and dissolved oxygen (**d**; n = 2 loggers) in the study site (Brewers Bay) during the month prior to and after Hurricane Irma. The vertical dashed lines correspond to Hurricanes Irma (Sep 6, 12:00 local time) and Maria (Sep 20, 02:00 local time), respectively.

**Figure 4:**
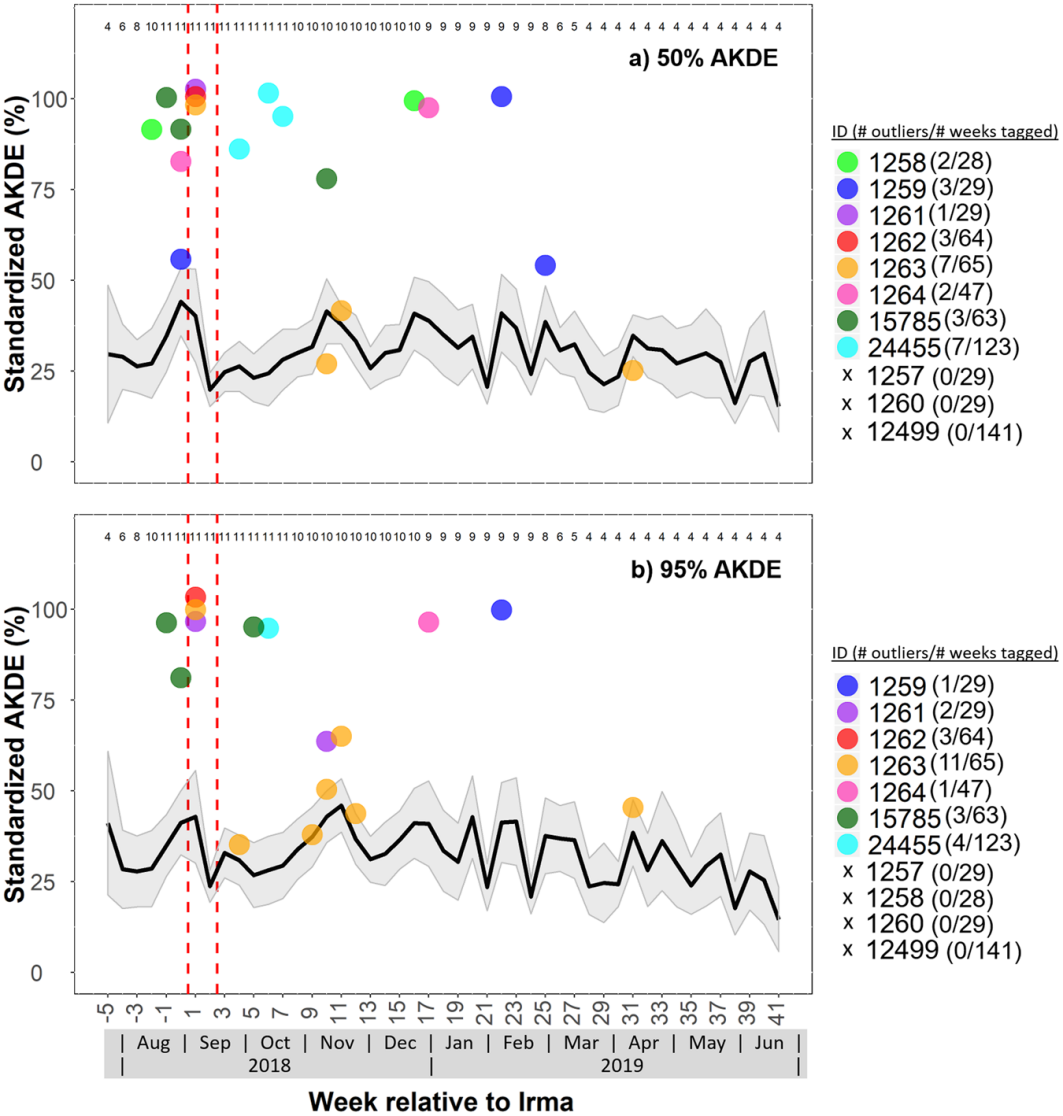
Mean 50% (**a**) and 95% (**b**) weekly auto-correlated kernel density estimates (AKDEs) of juvenile hawksbill turtles. The left-sided and right-sided red dotted vertical lines correspond to Hurricanes Irma and Maria, respectively. The grey shaded area represents the standard error of the mean. Weekly sample sizes (i.e., number of individuals) are indicated at top of plot. Only a subset of data (i.e., week −5 to 41 relative to Hurricane Irma) is plotted for clarity, and the total number of outliers throughout each individual’s detection period is summarized within the legend in parentheses. If no outlier occurred during the subset period an ‘x’ is marked in the legend for that individual. Outlier values do not exceed 100% but may appear so because overlapping points were scattered vertically (<4%) for clarity.

**Figure 5:**
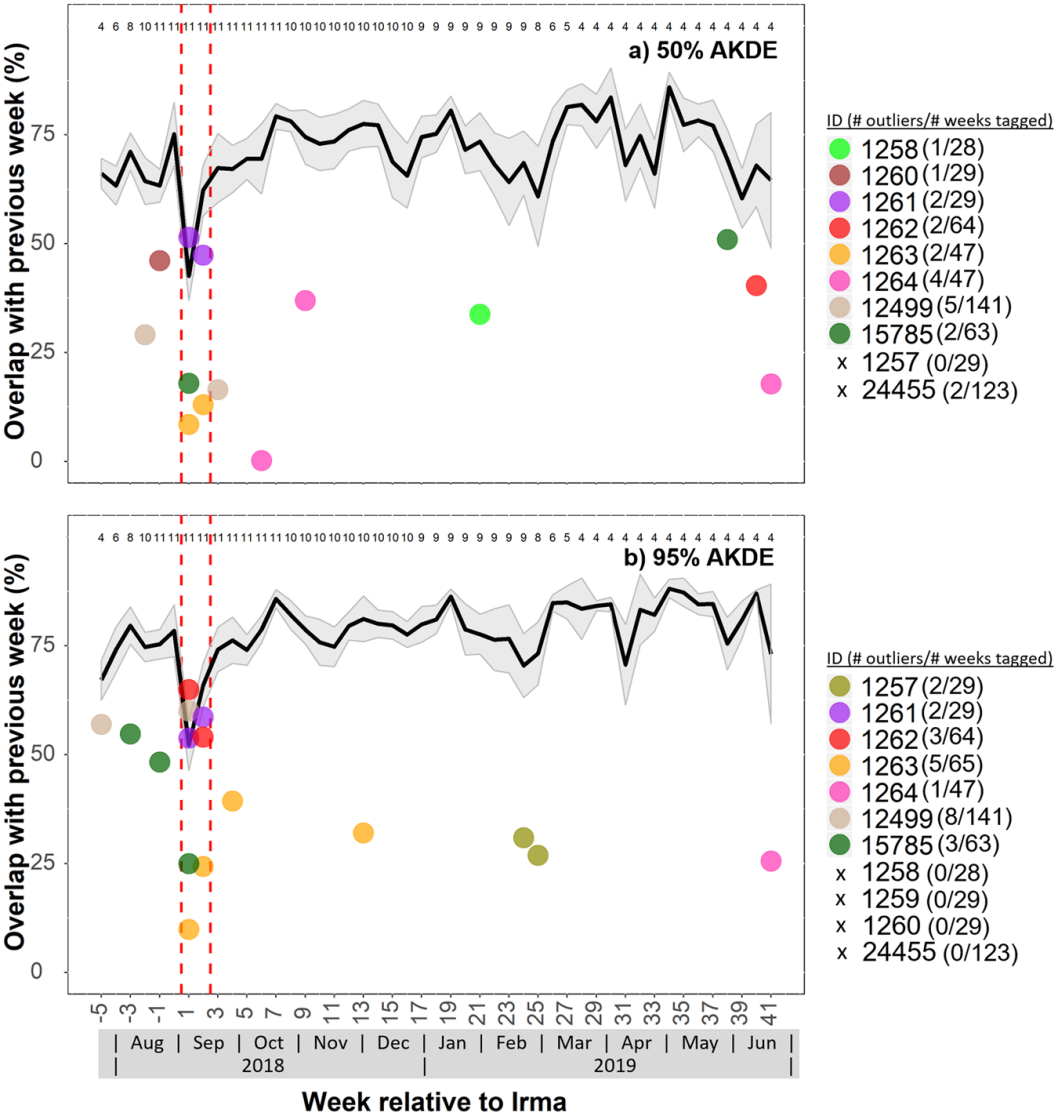
Mean 50% (**a**) and 95% (**b**) consecutive weekly AKDE overlap of juvenile hawksbill turtles. The left-sided and right-sided red dotted vertical lines correspond to Hurricanes Irma and Maria, respectively. The grey shaded area represents the standard error of the mean. Weekly sample sizes (i.e., number of individuals) are indicated at top of plot. Only a subset of data (i.e., week −5 to 41 relative to Hurricane Irma) is plotted for clarity, and the total number of outliers throughout each individual’s detection period is summarized within the legend in parentheses. If no outlier occurred during the subset period an ‘x’ is marked in the legend for that individual.

**Figure 6:**
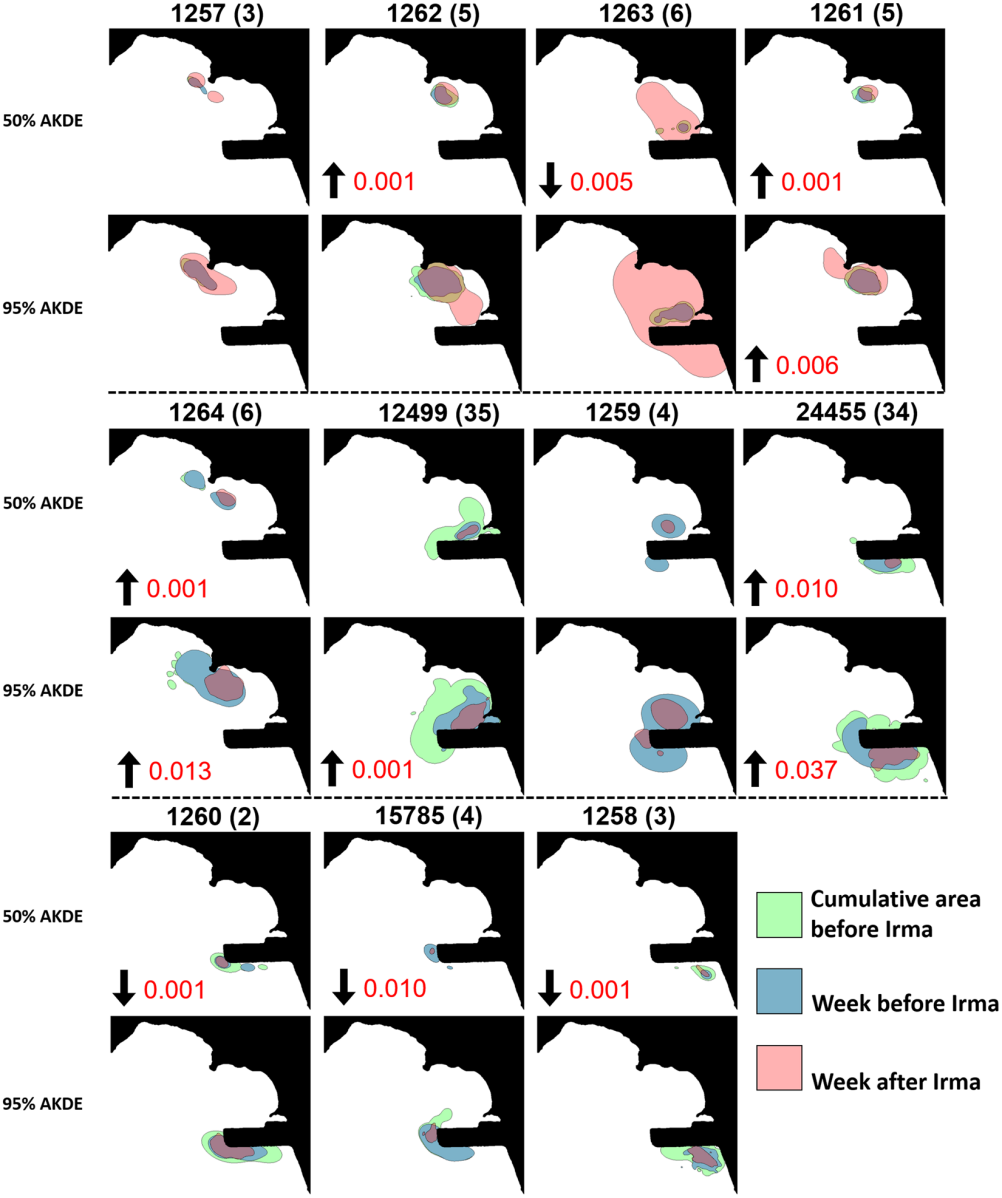
Cumulative weekly space-use plots of 50% and 95% AKDEs for juvenile hawksbill turtles including space-use one week prior to and after Hurricane Irma, as well as AKDEs from all weeks prior to Hurricane Irma combined. Each individual is identified at the top of each sub-plot; the numbers in parentheses represent the number of weeks prior to Hurricane Irma that individual had been tagged for. An arrow pointed down (and p-value next to it) refers to an increased use of deeper waters during the week after Irma (compared to the week before). An arrow pointed down indicates shallower waters were used following Irma.

A total of 11 hawksbill turtles had active transmitters attached when Hurricane Irma made landfall at Brewers Bay. Detections after June 2018 were removed from one turtle (1264) after it was identified to have dropped its tag. The total number of weeks turtles were tagged ranged between 29 and 140 weeks (Table 1), although some individuals were still being detected after October 2018. Most individuals had high weekly residency indices (i.e., 7/11 had mean values > 0.75), and there was no significant decrease in residency in Brewers Bay after Hurricane Irma both short-term (1–2 weeks) and long-term (>15 weeks; Fig. S1). Water temperature (~1 °C) and DO (~1–2 mg L^−1^) decreased abruptly during Hurricanes Irma and Maria (Figs 3; S2). The mean weekly space-use area estimates, 50% and 95% AKDEs, were ~0.046 km^2^ and 0.213 km^2^, respectively (Table 1). Standardized space-use sizes across individuals were relatively consistent throughout the detection period (Fig. 4), however, based on presence of outliers it was evident that several turtles (i.e., 1261, 1262, 1263) occupied larger core-use (50% AKDEs) and extent areas (95% AKDEs) during the week after Hurricane Irma, as well as the week prior to Hurricane Irma (50% AKDEs: 1259, 1264, 15785) compared to most other weeks. Similarly, outliers associated with reduced weekly space-use overlap were present during the week following Hurricane Irma for five of the 11 individuals (50% AKDE: 1261, 1263, 15785; 95% AKDE: 1261, 1262, 1263, 12499, 15785; Fig. 5). For some individuals the increase in space-use and decrease in weekly overlap after Hurricane Irma co-occurred with an increase in new space-use relative to past weeks tagged (i.e., cumulative overlap). For example, individuals 1261, 1262, and 1263 all used different areas immediately after Hurricane Irma compared to the cumulative area used five (or six) weeks prior (Fig. 6). This pattern was further demonstrated by the PCA that included the three spatial metrics and showed relatively large space-use area and new space-use area (and low space-use overlap) after Hurricane Irma for individuals 1257, 1261, 1262, and 1263 (Fig. S3). Three of these individuals (1257, 1261, 1262) were also mainly detected in the northern part of Brewers Bay (Fig. S3a), and two (1261, 1262) belonged to the smallest size class (<40 cm CL; Fig. S3b). Space-use metrics of the largest turtles (>50 cm CL) appeared to be less influenced by Hurricane Irma (Fig. S3b). The comparison of standardized cumulative overlap between individuals tagged in 2016 and 2017 demonstrated that home range size (or the maximum area used based on receiver coverage in Brewers Bay) was reached >15 weeks after tagging (i.e., when standardized cumulative overlap approached an asymptote; Fig. S4). Cumulative overlap of space-use extent (95% AKDE) increased at a greater rate in the turtles tagged in 2017 compared to 2016, whereas core space-use was similar between years (Fig. S4).

**Table 1:** Summary of sea turtles, fishes, and sting rays with active transmitters during Hurricane Irma.

ID	Common name	Size (cm) at tagging	Tag type	Date tagged	No. weeks tagged before Irma	Total no. weeks tagged	Mean (±se) no. of weekly position estimates	Mean (±se; km^2^) weekly 50% AKDE	Mean (±se; km^2^) weekly 95% AKDE
24455	Hawksbill turtle	28.1	V13P	Feb 2015	130	137	92 ± 5	0.039 ± 0.003	0.199 ± 0.010
12499	Hawksbill turtle	48.3	V16P	Apr 2015	125	140	157 ± 3	0.087 ± 0.003	0.347 ± 0.008
1257	Hawksbill turtle	44.5	V13P	Aug 2017	3	29	297 ± 10	0.043 ± 0.005	0.193 ± 0.019
1258	Hawksbill turtle	40.0	V13P	Aug 2017	3	28	186 ± 5	0.024 ± 0.002	0.134 ± 0.009
1259	Hawksbill turtle	40.5	V13P	Aug 2017	4	29	227 ± 11	0.052 ± 0.011	0.268 ± 0.048
1260	Hawksbill turtle	42.2	V13P	Aug 2017	2	29	298 ± 9	0.033 ± 0.003	0.16 ± 0.01
15785	Hawksbill turtle	53.2	V16P	Aug 2017	4	63	317 ± 5	0.017 ± 0.001	0.094 ± 0.004
1261	Hawksbill turtle	32.0	V13P	Jul 2017	5	29	305 ± 10	0.033 ± 0.001	0.154 ± 0.007
1262	Hawksbill turtle	36.0	V13P	Jul 2017	5	64	249 ± 5	0.054 ± 0.002	0.23 ± 0.007
1263	Hawksbill turtle	43.9	V13P	Jul 2017	6	65	274 ± 6	0.062 ± 0.008	0.28 ± 0.03
1264	Hawksbill turtle	35.5	V13P	Jul 2017	6	47	251 ± 7	0.058 ± 0.004	0.282 ± 0.014
	**Total**						**241 **±** 21**	**0.046 **±** 0.006**	**0.213 **±** 0.023**
45331	Southern ray	88	V13	Aug 2016	53	55	283 ± 4	0.050 ± 0.004	0.268 ± 0.019
45283	Southern ray	54	V9	Aug 2016	56	70	264 ± 5	0.043 ± 0.002	0.211 ± 0.007
24799	Southern ray	78	V13	Sep 2016	59	114	315 ± 4	0.023 ± 0.002	0.132 ± 0.009
45324	Southern ray	72	V9	Sep 2016	49	53	189 ± 7	0.062 ± 0.016	0.265 ± 0.043
45286	Lane snapper	28	V9	Aug 2016	54	70	313 ± 6	0.022 ± 0.003	0.115 ± 0.017
45291	Lane snapper	34	V9	Aug 2016	53	70	277 ± 9	0.043 ± 0.004	0.199 ± 0.013
59276	Mangrove snapper	44	V13	Mar 2016	75	122	185 ± 8	0.018 ± 0.003	0.132 ± 0.015
45339	Mutton snapper	39	V13	Sep 2016	48	91	331 ± 4	0.012 ± 0.001	0.052 ± 0.005
2966	Tarpon	85	V13P	Oct 2016	44	67	282 ± 9	0.236 ± 0.017	0.885 ± 0.052

Only data up to Oct 2018 is included (some transmitters may have been detected after this date). Sizes measurements for turtles, stingrays, and fishes are based on curved carapace length, total length, and fork length, respectively. Pressure sensors to measure depth were incorporated in tag type ending in ‘P’.

**Figure 7:**
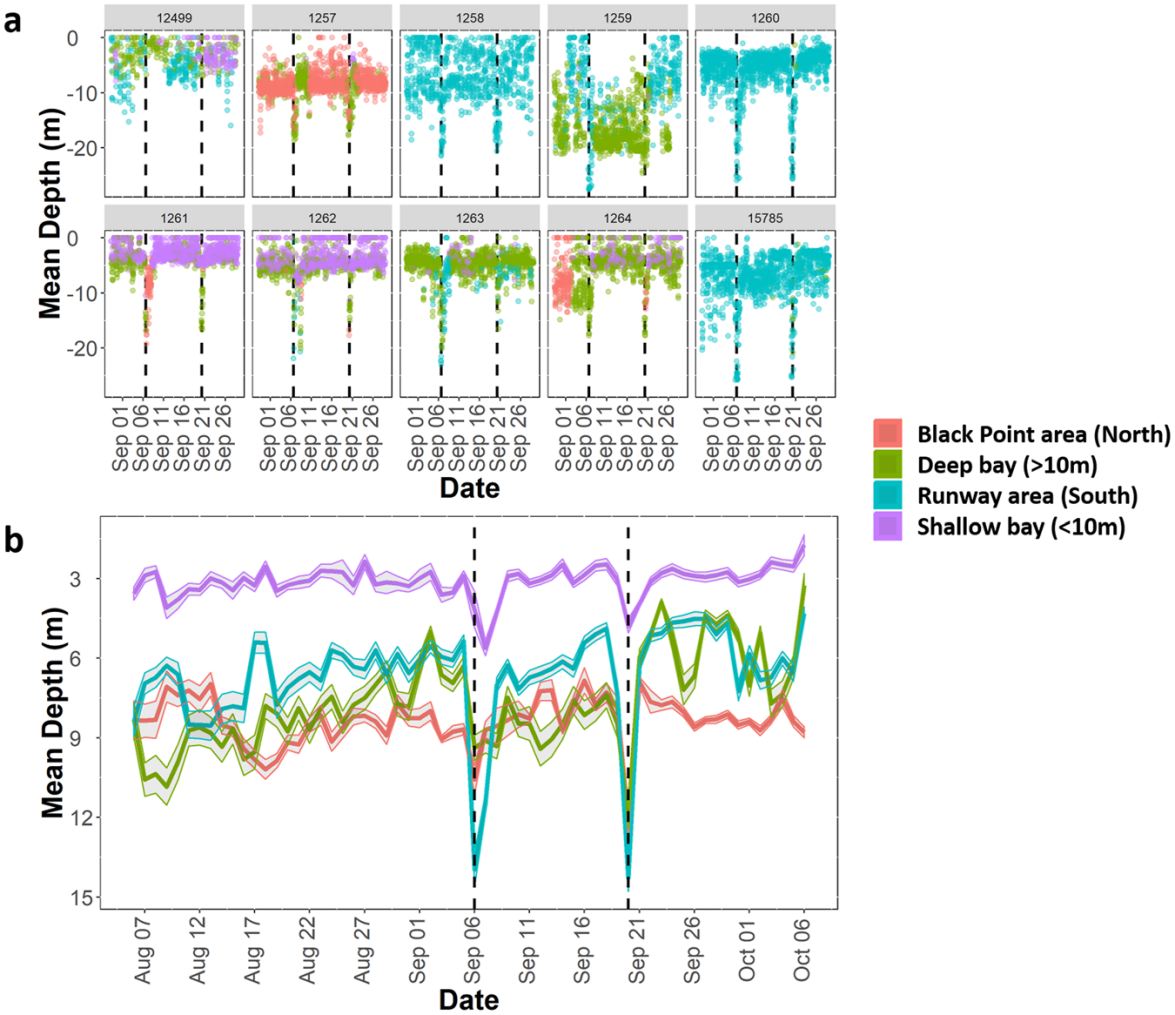
Mean 30-min centres of activity (COAs) depth-use for each individual juvenile hawksbill turtle (**a**) and mean daily depths for all individuals combined (**b**) during September 2017. Each coloured point/line corresponds to a specific area within Brewers Bay. The vertical dashed lines correspond to Hurricanes Irma (**a**: Sep 6, 12:00 local time; **b**: Sep 6) and Maria (**a**: Sep 20, 02:00 local time; **b**: Sep 20), respectively.

Investigation of movements immediately before, during, and after Hurricane Irma, showed a variety of patterns (Fig. S5). Turtles detected in the south (i.e., adjacent to the runway), remained localized to that area, whereas individuals further north were typically more mobile at these short-term periods. These patterns were also clear when depth-use was explored (Fig. 7a). Furthermore, there was an overwhelming trend of juvenile hawksbill turtles swimming deeper during both Hurricane Irma and Maria (e.g., 9/10 individuals tagged with depth sensors showed this behaviour; Fig. 7a,b). These vertical movements were often associated with horizontal movements or habitat shifts to access deeper waters. For example, turtles 1261 and 1262 left the shallow water area during the hurricanes (Fig. 7a). Alternatively, turtles 1258, 1260, and 15785 remained near the airport runway and increasingly used deeper water available there during the week after Irma (Figs 6 and 7a). Short-term (i.e., 32 hours) depth-use was significantly associated with the correlated parameters wind speed, atmospheric pressure, and water temperature during both hurricanes based on linear regressions (p < 0.05). Finally, there was no indication of significant decrease in body condition index for turtles that were recaptured in Brewers Bay up to a year after Hurricanes Irma and Maria (paired t-test, df = 6, p = 0.435).

### Fishes and rays

Six fish and four rays were tagged 44 to 75 weeks before Hurricane Irma hit Brewers Bay and were detected between 53 and 122 weeks total (Table 1). Residency varied among individuals with the mean number of weekly 30-min position estimates ranging from 185 (55%; mangrove snapper) to 331 (99%; mutton snapper) (Table 1). All individuals were detected >14 weeks after Hurricane Irma, except two southern stingray which were not detected in Brewers Bay more than four weeks after the first hurricane (Table 1). Furthermore, one of these southern stingrays was not detected following Hurricane Maria.

**Figure 8:**
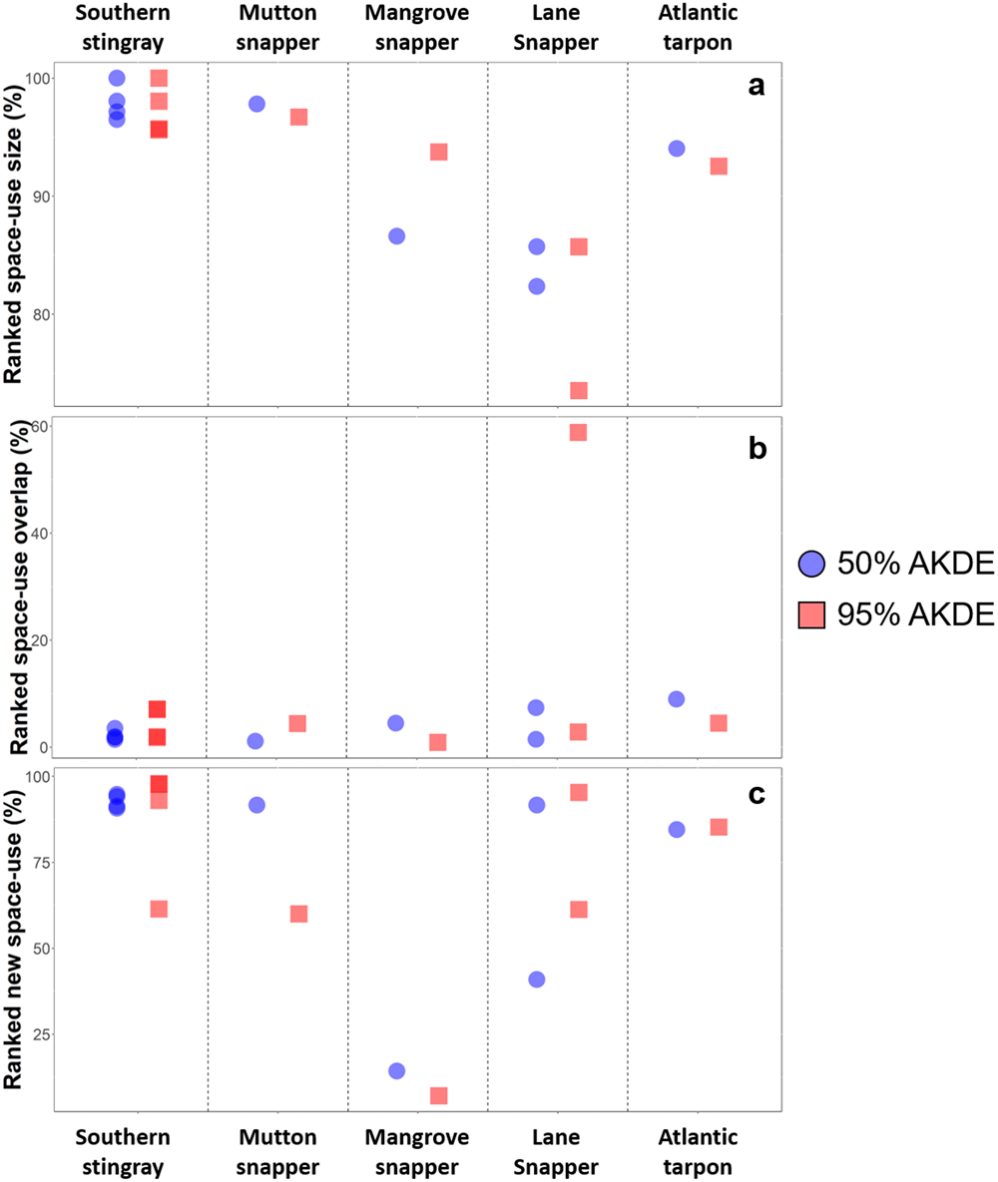
Spatial metrics (**a**: space-use size; **b**: space-use overlap, **c**: new weekly space-use) of stingrays and fishes during the week after Hurricane Irma (‘week 1’) estimated as a proportional rank (%) relative to the size of values in all other weeks (i.e., before and after Hurricane Irma). Each square/circle represents an individual. Note the y-axis scales vary.

**Figure 9:**
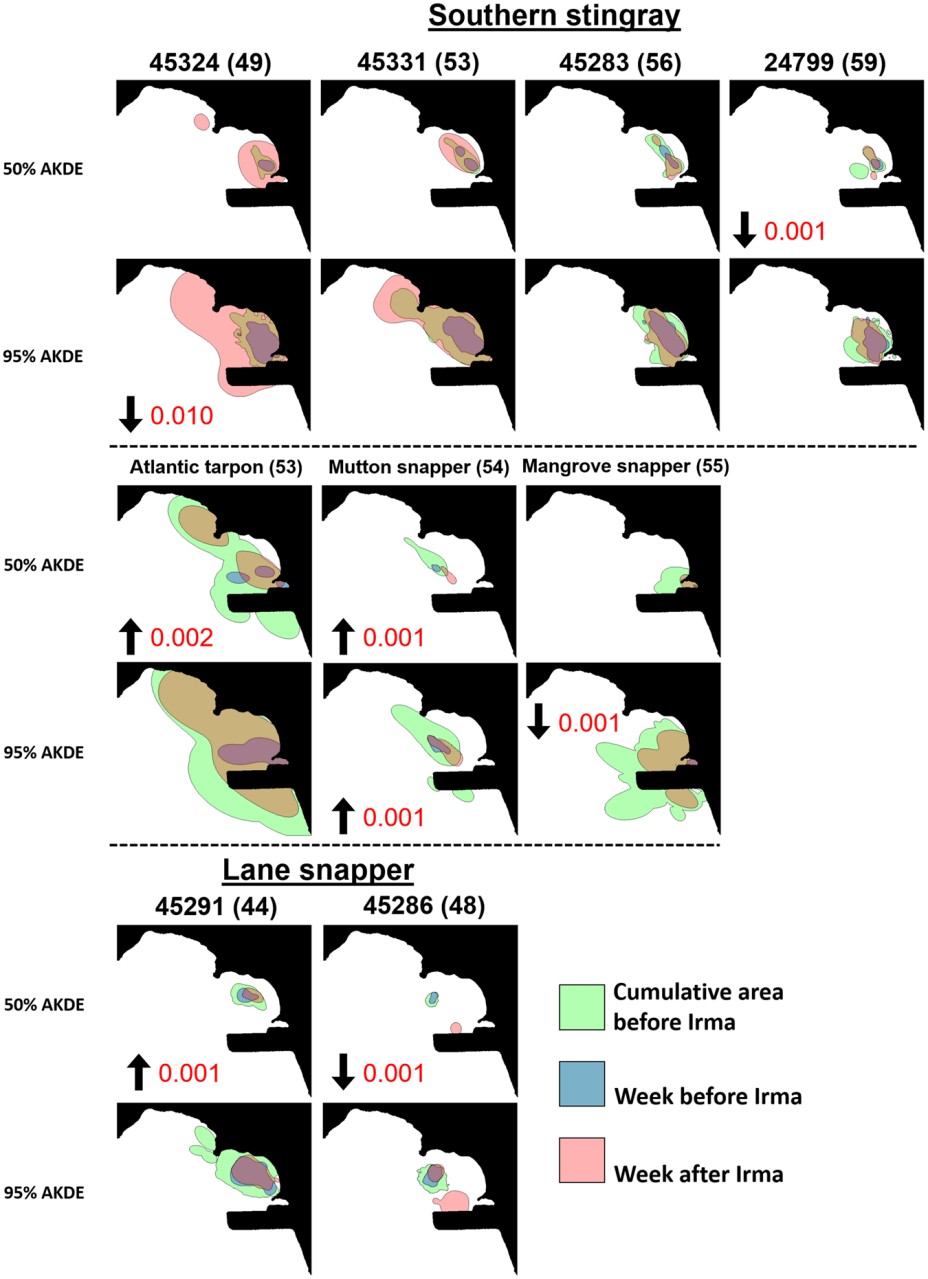
Cumulative weekly space-use plots of 50% and 95% AKDEs for fish and rays including space-use one week prior to and after Hurricane Irma, as well as AKDEs from all weeks prior to Hurricane Irma combined. Each individual is identified at the top of each sub-plot; the numbers in parentheses represent the number of weeks prior to Hurricane Irma that individual had been tagged for. An arrow pointed down (and p-value next to it) refers to an increased use of deeper waters during the week after Irma (compared to the week before). An arrow pointed down indicates shallower waters were used following Irma.

For fish and rays, when weekly spatial metrics (i.e., space-use size; space-use overlap, new weekly space-use) were ranked, the values during the week following Hurricane Irma were often relatively high (or low) compared to estimates outside this period (Fig. 8). Specifically, space-use size during the week following Hurricane Irma for all the tagged southern stingrays, mutton snapper, mangrove snapper, and tarpon were in the upper 85^th^ percentile for both 50% and 95% AKDEs compared to all other weeks detected (Fig. 8a). Similarly, they were also in the lower 90^th^ percentile for space-use overlap after Hurricane Irma compared to all other weeks (Fig. 8b). Ranked new space-use resulted in more variability among some individuals; for example, the mangrove snapper did not utilize new space immediately after hurricane Irma compared to southern stingrays, mutton snapper, and tarpon (particularly for 50% AKDEs; Figs 8c and 9). The new space exploited by southern stingrays during the week after Hurricane Irma differed among individuals but expanded outward, particularly to the north for the individuals (e.g., 45324, 45331) that had the largest area used (Fig. 9). The two lane snappers showed variable ranking for all three metrics (Fig. 8), as well as differences in activity space (50% AKDE) bottom-depth before and after Irma (Fig. 9); individual 45286 was unusual because it shifted its activity space to a completely new habitat in deeper water adjacent to the airport runway after Hurricane Irma (Fig. 9). A PCA was not possible among spatial metrics due to lack of discrimination among variables/individuals.

Distinct short-term movement patterns were apparent for at least six of nine individuals. For example, three of the four rays and some fishes (e.g., lane snapper - 45291, tarpon, mangrove snapper) moved westward toward deeper water areas in Brewers Bay during Hurricane Irma (Fig. S6). One ray (24799) moved to the southwest during the hurricane to deeper water along the airport runway (Fig. S6). No discernible depth pattern was observed from the pressure sensor in the tarpon (the only fish with this sensor incorporated), although it was detected more readily in the Black Point area to the north immediately after Irma (Fig. S7) using shallower waters (e.g., 50% AKDE, Fig. 9).

## Discussion

Two of the largest hurricanes in recorded history devastated St Thomas, USVI (United States – Virgin Islands) and nearby islands less than two weeks apart in September 2017. Brewers Bay was hit with powerful winds (~287 km h^−1^), large waves and storm surges several feet above sea level. It resulted in the destruction of surrounding university and airport buildings on-land, and significantly disturbed habitats in-water (e.g., seagrass beds were decimated, coastal areas eroded, and siltation increased during/after each hurricane; JKM pers. obs.), the long-term effects not yet known. Every hawksbill turtle remained in Brewers Bay for at least six months to over a year after these hurricanes and demonstrated specific adaptive behaviours in response to these disturbances such as short-term expansions of activity space and deep movements. Similarly, most fishes continued to utilize Brewers Bay at least several months after the hurricanes despite changes in behaviour immediately following Irma. However, it was evident that southern stingrays were significantly impacted (e.g., by food/habitat availability or physiological constraints), and three of four tagged individuals left Brewers Bay after the hurricanes (between 2–14 weeks after Irma). Generally, Hurricane Irma caused stronger responses in animal behaviour compared to Hurricane Maria – a slightly less severe storm whose path (and resulting storm surge) was further from our study site.

### Hawksbill turtles

As juveniles, hawksbills commonly inhabit shallow bays, mainly for protection and physiological benefits^26,27^ (e.g., access to food). Given the high residency in Brewers Bay before and after Hurricane Irma, there appears to be adequate resources despite the disturbances. This is supported by similar body conditions among recaptured individuals following the hurricanes. Juvenile hawksbills consume a variety of prey including algae, marine plants, sponges and other invertebrates^28^, which may make them more resilient to disturbance compared to other turtle species. For example, green sea turtles (*Chelonia mydas*) were not observed in Brewers Bay for several weeks after Irma removed most of the shallow seagrass beds – their primary food source^29^. A major research priority is to better understand juvenile sea turtle foraging grounds, as well as the impact of environmental disturbances on habitat^30^; this study highlights that the critically endangered juvenile hawksbill is highly dependent on a small area long-term to obtain necessary resources even after a major disturbance.

Outliers from the three weekly metrics (activity space size, overlap, and cumulative/new) used to explore spatial changes relative to the hurricanes indicated that several turtles responded to the disturbances by either increasing the size of their space use (3/11), shifting their activity space to different areas (5/11), or occupying locations never before visited (5/11) after Hurricane Irma. The application of outliers was a useful tool to highlight variation associated with extreme data points relative to spatial patterns throughout each individual’s detection period. We acknowledge that the occurrences listed above relative to Hurricane Irma are not necessarily causative and that other outliers occurred outside this time period. Nevertheless, given the relatively few number of outliers across extensive individual detection periods (between 28–140 weeks) compared to those during the week following Irma, the results strongly suggest Hurricane Irma significantly influenced hawksbill behaviour short-term. Caution should also be taken when interpreting the cumulative/new activity space results because most individuals were tagged <6 weeks prior to Hurricane Irma, a period in which the total cumulative activity space was not yet reached. Consequently, it is difficult to differentiate whether the hurricane(s) caused increases in cumulative activity spaces (i.e., cumulative AKDEs in 2017) or if it was an artefact of ‘normal’ movement patterns after release (i.e., cumulative AKDEs in 2016). The former case seems more plausible, especially for activity space extent (95% AKDEs) given the steeper rise in AKDEs between ‘week 2’ and ‘week 6’ since tagging in 2017 (i.e., during Hurricane Irma) compared to turtles tagged in 2016.

There are several possible reasons for a short-term (weekly) change in activity space caused by Hurricane Irma; for example, increased foraging effort may have been necessary to meet energetic requirements after the disturbance. This behaviour has been summarized by Sergio *et al*.^1^ and includes examples of birds, bats, reptiles, and mammals increasing foraging ranges following environmental disturbances. Interestingly, it was the largest individuals that refrained from changing their activity space after Hurricane Irma. This could indicate that larger individuals are more resilient to disturbances whether it from increased energetic reserves, competitive ability, learned experience, or pre-disturbance site selection^2,31^. Alternatively, new sites for protection or resting may have been exploited^32,33^. The individuals that readily displayed increased activity space (and new space-use) predominantly inhabited the northern portion of Brewers Bay; consequently, this area could have been affected more than other locations resulting in diminished resource availability or adequate protection. However, it is difficult to be certain about locale-specific effects because receiver coverage was restricted in some locations, specifically on the west and south sides of the runway where only a line of receivers was positioned (i.e., less spatial heterogeneity). There was evidence that a few turtles (3/11) increased their space use the week prior to Hurricane Irma. It is not uncommon for animals to alter space use patterns in response to changing atmospheric pressure (i.e., atmospheric pressure dropped as the hurricanes approached)^34–36^. For example, Liu *et al*.^37^ reported the departure of sea snakes (*Laticauda* spp.) from a littoral habitat in Taiwan prior to typhoon Morakot, which corresponded with dropping atmospheric pressure as opposed to precipitation or windspeed. Although no turtles left Brewers Bay prior to the hurricanes, increased space use may have been a responsive or preparatory behaviour to the approaching hurricane.

It was also evident that the hurricanes had an even more immediate impact on the behaviour of juvenile hawksbill turtles in Brewers Bay. Specifically, during both hurricanes, every individual, except one (which only had two detections during the hurricane), moved to deeper water – sometimes twice as deep than routine. This behaviour never occurred outside of these periods during the month of September, supporting it as a response to the environmental disturbance, and has been reported in other marine reptiles^37^, fishes^38^, and elasmobranchs^35^. Deeper dives corresponded to the abrupt changes in water temperature, wind speed, and atmospheric pressure, but it is unclear which factor(s) was most critical as they were all correlated during the storm. We hypothesize that deeper water was utilized as a direct response to the physical disturbances in the water because this behaviour was only instigated once wind speeds rose above a certain level only a few hours before the peak of the storm passed St. Thomas. For several individuals, there was a shift in spatial patterns as well. For example, sea turtle 1257 primarily made the deeper dives in the deeper section of Brewers Bay, where as the remainder of September it was in shallower areas mainly at ‘Black Point’. Not surprisingly, most deep dives were made in the ‘deep’ and ‘runway’ portions of Brewers Bay, which are in or adjacent to the deepest areas within the receiver array. The movement tracks during Hurricane Irma demonstrate several individuals moving to these new deeper areas once the storm began. As previously mentioned, the individuals towards the south of the runway appeared to move less during and immediately after Irma, however less receiver coverage existed to track individuals in this area (which is adjacent to water >20 m deep). Dive times during the storm events could not be reliably calculated due to reduced receiver efficiency.

### Fishes and rays

The most consistent pattern of spatial change from the hurricane was displayed by southern stingrays that expanded their activity space outward after Irma. Of particular interest were three individuals that had the greatest change in activity space and ceased to be detected in Brewers Bay a few weeks later. Prior to the hurricanes, each stingray was consistently detected nearshore within the array for approximately one year, indicating long-term residency (see also ref.^39^). The southern stingray is commonly identified as a generalist epibenthic and infauna consumer of invertebrates (e.g., annelids, decapods, and polychaetes) and small fishes^40–42^. It is unclear what impact the strong winds and oceanographic changes had on the shallow water prey of the tagged individuals. However, it was observed that large areas of shallow soft bottom habitats (sand and seagrass) were scoured over a meter deep exposing long buried ancient reef structures (RSN pers. obs.), which may have altered prey availability or foraging habitats, driving these individuals to deeper water outside the array or to less damaged areas. Driven by deep-water mixing during the two hurricanes, the rapid declines in water temperature of nearly 2 °C (~30–28 °C) may have disrupted metabolic functioning, as suggested by Tobin *et al*.^43^ for fishes in the Great Barrier Reef after a cyclone, although this change was within seasonal thermal fluctuations year-round (~26–30 °C). Dissolved oxygen levels, which dropped over 20% in both shallow and deep areas (by ~1–2 mg L^−1^) and remained low after several weeks, may have also been a contributing factor for several rays leaving the bay. Indeed, Schlaff *et al*.^44^ provide a summary of environmental variables attributed to influencing elasmobranch behaviour, including examples of animals avoiding hypoxic (reduced DO) areas (see also refs^45,46^).

Individual behaviours for fishes and rays in response to Hurricane Irma revealed important preliminary information concerning the effects of extreme environmental occurrences. Most notable was the majority of individuals demonstrated higher activity space size and new activity space areas immediately following Hurricane Irma, in combination with low overlap in space use between consecutive weeks. Only a few fish fell outside the upper (or lower) 75% percentile measurements. These acute changes in behavior due to a passing hurricane are in contrast to fishes that are displaying reproductive behaviours. For example, Locascio & Mann^47^ and Biggs *et al*.^48^ showed considerable resilience to hurricanes in two species of Sciaenidae during spawning. Both *Cynoscion arenarius* and *Cynoscion nebulosus* continued producing courtship-associated sounds during peak storm conditions. In both cases, however, the seatrout shifted their chorusing a few hours earlier for several days after the hurricane.

Although noise disturbances during the hurricanes limited our ability to track fine-scale movements, several trends were apparent. Based on the short-term movement tracks, several species (including southern stingray, lane snapper, tarpon, and mangrove snapper) moved away from shallow nearshore areas during Irma to deeper waters. For example, the activity space of the juvenile tarpon, an inshore/pelagic inhabitant in Brewers Bay, was relatively large compared to other weeks, although limited new area was utilized due to the highly mobile nature of this species^49^. The mangrove snapper was detected leaving the shallow lagoon area during Irma while all the southern stingrays shifted their locations to the west towards the deeper waters of Brewers Bay. Presumably, this behaviour was to seek refuge from the turbulent surface waters or as an innate flight response^34^. For example, in a recent study, Bacheler *et al*.^13^ reported that during passing hurricanes grey triggerfish greatly increased their rates of movement and emigration to deeper water from a deep reef (37 m) primarily in response to wind-generated wave velocity.

## Conclusion

The results from this study were collected opportunistically in relation to two unpredictable and destructive storm events, thus the sample size, tagging regime (i.e., hawksbills had only been tagged a few weeks before Irma), and receiver spatial coverage (i.e., due to loss of three receivers and acoustic interference during the storm) were not optimally designed. Nevertheless, the reporting of behaviour prior to and following such a catastrophic occurrence are extremely rare and our results provide novel information concerning a variety of species-specific responses within a coastal community. Furthermore, the longevity and spatial resolution of detections provides a surfeit of baseline behavioural data to compare with the disturbance events and demonstrates the overwhelming short-term impacts hurricanes have despite their irregularity.

Animals respond to environmental disturbances in a variety of ways through changes in behaviour, demography, genetics, life history, and physiology, all of which can affect community organization^1^. The immediate effects of the hurricanes were evident by the movement of species to deeper waters. It was also apparent that species with small activity spaces in Brewers Bay (e.g., lane snapper and mutton snapper) shifted locations during Irma, likely to find refuge. These direct responses did not last long, however during the week following Hurricane Irma, activity spaces often shifted and expanded, suggesting short-term effects on the ability of animals to find food or other necessary resources. Apart from increased hawksbill turtle depth-use during Hurricane Maria, there was limited evidence that animals changed spatial patterns following this hurricane. Maria was less powerful than Irma on St. Thomas, resulting in heavy rainfall but less extreme wind speeds. It is probable that habitat loss and specific disturbances were already optimized after Hurricane Irma leading effects of Maria to be minimal, except in cases where individuals could not meet specific requirements in Brewers Bay, such as southern stingray. Overall, the long-term effects of both Hurricane Irma and Maria appear to be limited. The majority of species (besides stingrays) returned to the areas frequented prior to the hurricanes and demonstrated consistent activity spaces through time. Spatial patterns are not the only indicator of resilience to disturbances; therefore, we cannot comment extensively on the long-term implications on physical condition, energetics, or nutrition, but post-hurricane body conditions of turtles suggested a healthy population. For at least one species, the southern stingray, it was evident that the hurricanes disrupted long-term behaviour, suggesting they are particularly vulnerable to these extreme environmental conditions. The consequences of leaving established areas could be severe unless new reliable food sources are discovered, and protection from predators or harmful environmental conditions (e.g., temperature, oxygen, salinity, etc.) are established. The strong site fidelity, despite catastrophic storm activity, shown by the hawksbill turtles reveals the importance of established juvenile foraging grounds for this critically endangered animal. However, if severe storms or hurricanes become increasingly frequent, habitat destruction and loss of valuable resources may further prevent recovery from deleterious disturbances particularly for those species with limited resilience or specialized resource niches.

## Methods

### Study area and acoustic telemetry sampling

The effect Hurricane Irma and Maria had on animal behaviour was investigated using an existing acoustic telemetry array in (and around) Brewers Bay, St. Thomas, US Virgin Islands (18.3425°N, 64.9800°W; Fig. 2). Brewers Bay is a small bay (~1 km^2^) located adjacent to the University of the Virgin Islands and the Cyril E. King International Airport in the southwest portion of the island. The habitats within Brewers Bay (and adjacent areas) mainly consist of seagrass, sand, fringing coral and rocky reefs, patch reefs of corals and sponges, and artificial concrete structures around the airport runway. Given the small-scale variability in habitat composition, these areas were broadly categorized into four groups: shallow (<10 m bottom-depth), deep (>10 m bottom-depth), Black Point (5–30 m bottom-depth; the northern point within Brewers Bay), and runway (the southern part of Brewers Bay; Fig. 2). The artificial habitat around the runway provided both shallow foraging habitat but also dropped steeply into water 10 to 30 m deep. The array was deployed in 2015, typically consisting between 35 and 43 Vemco^©^ VR2W-69 kHz receivers, moored near the bottom using rope and concrete blocks or sand screws in shallow soft bottom habitats. The detection range in Brewers Bay varied between habitat and tag type and based on short-term (~one week) range tests in June 2015 including receiver-transmitter distances between 25–400 m, range was estimated between ~70–100 m, 90–110 m, and 140–230 m for V9, V13, and V16 Vemco^©^ transmitters, respectively (75% probability; Table S1; JKM pers. obs.), representing ~2 km^2^ of total receiver coverage across the whole array. Temperature (U22-001 HOBO^®^ Water Temp Pro v2; Onset Computer Corporation, Bourne, MA, n = 12) and dissolved oxygen (DO; RS232 miniDOT^®^ logger, Precision Measurement Engineering; Vista, CA, n = 2) were collected at several sites within Brewers Bay. Wind speed and atmospheric pressure obtained from NOAA National Data Buoy Center for buoys near adjacent islands St. John (station 41052), USVI and Vieques (station 41056), Puerto Rico (https://www.caricoos.org/?locale=en) were included to highlight meteorological conditions around the hurricanes.

The following animals were tracked within the Brewers Bay array with acoustic transmitters prior to and after Hurricane Irma (Table 1): juvenile hawksbill turtle (*Eretmochelys imbricata*; n = 11), juvenile southern stingray (*Hypanus americanus;* n = 4), lane snapper (*Lutjanus synagris*; n = 2), mangrove snapper (*Lutjanus griseus*; n = 1), mutton snapper (*Lutjanus analis;* n = 1), and a juvenile Atlantic tarpon (*Megalops atlanticus*; n = 1). The battery life of all transmitters extended into 2018. The main species tracked was the hawksbill sea turtle, a critically endangered species that has suffered ~80% population decline over the last 105 years^50^, and ≥95% population decline in Caribbean populations^51–53^. As juveniles, hawksbills recruit to nearshore reef habitats and reside locally for long-term periods^26^. Past research has demonstrated the debilitating effect of hurricanes on hatchling survival and nesting quality^54,55^, but no research has described small-scale spatial patterns of free-swimming individuals in relation to extreme weather events. Southern stingray, a subtropical demersal mesopredator typically inhabiting shallow beach, seagrass, lagoons, or reef habitats, was also tracked in this study. Limited information is available concerning the movement patterns of this common coastal species, particularly for extended periods of time. Similarly, their conservation status is ‘data deficient’^56^ indicating more research is broadly needed to assess populations, distribution, vulnerabilities, and resilience to natural or anthropogenic disturbances. The three snapper species, all known to make seasonal spawning migrations or participate in fish spawning aggregations outside Brewers Bay^57^, had smaller sample sizes, but were detected long-term, and thus were included. Similarly, a juvenile tarpon, a pelagic consumer inhabiting coastal waters often near the surface, was included in behavioural explorations.

For the hawksbill turtles, individuals were caught in Brewers Bay by hand on snorkel, and transmitters were attached to two marginal scutes (adjacent to the postcentral scutes) via plastic coated wire (after drilling two small holes at the extremity of the scutes) and marine putty (Fig. S8). The stingray and fishes were caught by hook and line, placed in fresh seawater and were turned onto their dorsal surface to induce tonic immobility^58^. The transmitter was surgically implanted in the gut cavity (ventral and adjacent to midline) with sterilized scalpel and forceps and the incision was closed with 2–3 chromatic gut or silk surgical sutures or staples. Surgical procedures were carried out with approval and in accordance to relevant guidelines from the University of the Virgin Islands (IRBNet ID: 747807-3) as approved by US National Marine Fisheries Service protected species permit #15809. Morphometrics (e.g., mass, carapace length, fork length, sex) were taken prior to surgery and all animals were released within the Brewers Bay array.

### Data analysis

Receiver data were compiled for hawksbill turtles, fishes, and rays that were detected for at least two weeks prior to Hurricane Irma. All detections for these individuals were used for further analyses, unless otherwise stated below. After Irma, 3 of 43 receivers in Brewers Bay were lost and replaced. To ensure detections were not biased by the loss or replacement of receivers throughout the array deployment period, only receivers that were consistently deployed (and present after animals were tagged) were used in this study (n = 34 receivers). Raw detection plots were explored to inspect whether transmitters were dropped from turtles, or fishes/rays had died within the receiver array. If detections occurred consistently on only one receiver (or a small cluster of receivers with overlapping detection ranges) without any distinct behavioural pattern, the affected detections were removed.

Pursuant to the main goal of this study, space-use was estimated prior to and after Hurricanes Irma and Maria. Estimates of space-use calculated at weekly intervals were determined to be the minimum temporal resolution in which enough detections were obtained to accurately estimate spatial trends. For example, daily spatial estimates were highly variable, particularly for fishes that were not detected as consistently as hawksbill turtles or southern stingrays. Due to the catastrophic impact of Hurricane Irma compared to Hurricane Maria (at least on the island of St. Thomas), weekly spatial estimates were temporally centered around Hurricane Irma. Specifically, detections from the day that Hurricane Irma hit St. Thomas (Sep 6, 2017) were not included to ensure that reduced receiver efficiency from wind noise, large swells and water turbidity did not influence spatial estimates^35^. As a result, ‘week 0’ consisted of detections between Aug 30 - Sep 5, and ‘week 1’ included detections between Sep 7–13. Hurricane Maria passed through St. Thomas the night/morning of Sep 19/20; consequently, ‘week 2’ represents the week Hurricane Maria hit Brewers Bay and ‘week 3’ is the following week.

Weekly space use was estimated using autocorrelated kernel density estimates (AKDEs) described in refs^59,60^. This approach is advantageous compared to traditional kernel density estimates (KDEs) because it accounts for non-stationary, autocorrelated, and continuous movement processes common with acoustic telemetry data. Prior to implementation of AKDEs, the location of each detection was estimated based on probability distributions relative to the distance away from a receiver. Previous range tests in Brewers Bay were utilized to estimate the probability of a detection occurring up to 400 m from a receiver based on four different habitat/bottom compositions (Table S1). To accommodate uncertainty with animal locations (i.e., within the range of the receiver), each detection was placed at a random direction and distance weighted by the habitat-specific (and transmitter-specific) probability curves. If this new location was placed on-land, it was re-located to the receiver location. Next, centers of activity^61^ (COAs) were implemented at 30-min intervals to further assist with spatial and temporal autocorrelation prior to use of the kernel density estimator (i.e., mean-weighted 30-min positions will be less correlated in space and time than consecutive 3-min receiver locations). Using a mean-weighted position approach also restricted the effect of false detections (i.e., a false detection, although rare, will have a smaller impact when pooled with other detections). The 30-min time interval was selected based on guidelines provided by Simpfendorfer *et al*.^61^ in which the number of data points and unique positions are both optimized based on the time interval chosen. If a COA location was placed on-land it was re-located to a random location within the minimum convex polygon of all detections for that 30-min period. After a preliminary data exploration, only weeks with a minimum number of 15 and 25 COA positions were chosen for fishes/rays and turtles, respectively, as cut-offs to avoid calculating AKDEs from weeks with limited data. The R packages ‘move’^62^ and ‘ctmm’^59^ were used to calculate AKDEs. The optimally weighted OUF (Ornstein-Uhlenbeck-F) model with continuous velocity motion (derived from Ornstein-Uhlenbeck (OU) process) and built-in autocorrelation function was fit to the weekly COA data for each individual. The total areas for 50% and 95% utilization distributions (UDs) were calculated for each weekly AKDE to represent core areas and range extent areas used, respectively. If 50% or 95% UDs overlapped with land, that portion of area was clipped from each overall estimate.

Two approaches were used to examine whether new or different locations in Brewers Bay were used after Hurricane Irma. The first method determined the cumulative area of 50% and 95% UDs each week after tagging. Consequently, for example, if during ‘week 1’ (i.e., the week after Hurricane Irma) the cumulative area increased, then new locations/areas were utilized by an individual that week compared to all preceding weeks since tagging. The second method incorporated the spatial overlap between consecutive weeks. For example, if the area of overlap decreased between ‘week 0’ and ‘week 1’ (i.e., before and after Hurricane Irma), then different locations/areas between weeks were used. The area of overlap was calculated as the overlap area of week *n* with week *n -1*, divided by the average area of week *n* and *n -1* combined. For weeks that did not meet the minimum requirement of detections, the cumulative and overlap area deriving from the previous week was used (or the next prior week that had enough detections). Cumulative areas for the nine hawksbill turtles (analyzed individually) tagged <5 weeks prior to Hurricane Irma were compared with four other individuals tagged during the equivalent time period in the year prior (2016) to evaluate whether increases in cumulative area, particularly in recent weeks after tagging, may have been driven by typical home range distribution/expansion after tagging, as opposed to altered behaviour due to hurricanes (e.g., greater space-use in consecutive weeks may result from normal behaviour affiliated with home range size). Weekly residency indices were also calculated to evaluate whether individuals reduced their use of Brewers Bay after Hurricane Irma. These were calculated as the number of observed weekly COAs divided by the number of weekly 30-minute COAs possible (i.e., 336 week^−1^). Finally, mean depths were calculated for each COA period to investigate whether depth patterns changed as a result of Hurricanes Irma and Maria.

The magnitude of Hurricane Irma produced wind speeds and wave heights several orders higher than normal in a very short time and therefore provided a unique opportunity to examine acute changes in the target animals’ normal behavior. As a result, the presence of outliers (±1.5*inter-quartile range) was used to indicate the weekly time periods when individual spatial estimates (i.e., AKDE area, overlap area, cumulative area, residency index) were higher or lower than typical throughout the detection period. This method was also deemed more informative than commonly used approaches in acoustic telemetry (e.g., generalized mixed effects models) since behavioural responses to these rare and brief environmental events varied among individuals (and species), therefore an individual-based approach to interpret the quantitative spatial metrics with the hurricanes as focal points was preferred. In addition to identifying weeks with individual outliers, principal component analysis (PCA – with covariance matrix) was used to visualize the relationship among individual hawksbill turtles and six spatial metrics (AKDE area - 50% and 95%, overlap area - 50% and 95%, cumulative area - 50% and 95%) in multivariate space. The spatial metrics for each individual were standardized as weekly ranks (as a proportion) to enable comparison among variables. Following this, only ‘week 1’ values were included in the PCA to specifically evaluate the influence Hurricane Irma had on space-use directly after. Spatial metrics with high correlations were removed from the PCA to avoid overweighting redundant variables. All turtles were grouped by size category (<40 cm, 40–50 cm, >50 cm; curved carapace length - CCL) and location of occurrence after Hurricane Irma (i.e., center of 50% AKDE in North, South, or middle of Brewers Bay) to explore potential behavioural commonalities associated with the spatial metrics. Due to difference in tagging dates, the CCL grouping described above was based on size estimates for each individual at the time of Hurricane Irma using growth curves from Hawkes *et al*.^63^. Due to the few numbers of fish and rays tagged at the time of Hurricane Irma, and because some individuals were not detected long-term following the hurricanes, the values of weekly spatial metrics were ranked relative to Hurricane Irma as opposed to outlier occurrences.

Due to the destruction of many weather stations and nearby oceanographic buoys caused by Hurricane Irma, many environmental parameters (e.g., wind speed and air pressure) during and several weeks after could not be reliably collected on St. Thomas. However, water temperature and DO data collected in Brewers Bay from loggers that survived the hurricanes, and atmospheric pressure and wind speed from oceanographic buoys near adjacent islands (St. John and Vieques), helped describe trends in behaviour associated with the hurricanes. Linear regressions tested whether these environmental parameters influenced sea turtle depth-use (using 30-min average depth measurements) immediately before (4 hrs), during (24 hrs), and after (4 hrs) the hurricanes were closest to St. Thomas. To explore whether deeper areas were used after Hurricane Irma, 100 bottom-depth values were randomly selected within the AKDE areas during the week prior to and the week after Irma. Differences in bottom-depth values between the two weeks were tested with a Mann-Whitney U test (independent samples); a non-parametric approach was used because bottom-depth distributions were often right-skewed prior to Irma and left-skewed following Irma, making transformation difficult. Finally, to test if there was a declining trend in body condition (a proxy to health) among sea turtles after the hurricanes, a paired two-tailed t-test comparing Body Condition Index (BCI = mass/curved carapace length^3^ × 10^4^) values of seven turtles that were captured before and after the hurricanes was used. Alpha was set at 0.05 as a potential indicator of significance for the three tests above, although lower values are reported for better resolution. All data analysis was conducted in R version 3.4.1^64^.

## Supplementary information

Supplementary material

## Data Availability

The datasets generated during and/or analysed during the current study are available from the corresponding author on reasonable request.
