# Supplementary material for: Vulnerability of sea turtles and fishes in response to two catastrophic Caribbean hurricanes, Irma and Maria
Source: Sci Rep. 2019 Oct 3;9:14254. doi: 10.1038/s41598-019-50523-3 (PMC6776526; doi:10.1038/s41598-019-50523-3)
Supplement: Supplementary file 1 — Supplementary material [file 41598_2019_50523_MOESM1_ESM.pdf]

Supplementary Materials

Title:

Vulnerability of sea turtles and fishes in response to two catastrophic Caribbean hurricanes, Irma and Maria

Authors:

JK Matley[1*,2], S Eanes[2], RS Nemeth[2], PD Jobsis[2]

Table Supplementary 1: Habitat- and transmitter-specific parameters used to estimate detection probabilities for the use in AKDEs. Range tests were completed during ~one week (Jun 2015) and included receiver-transmitter distances in each habitat (one transmitter/six receivers per habitat) between 25 – 400m.

Tag type	Power (db)	Habitat type	Slope (logged)	Y-intercept	Distance (m) at 75% detection probability
V9	151	Shallow coral/rock	-0.481	2.842	77
		Shallow sand/seagrass	-0.435	2.672	83
		Deep coral/rock	-0.445	2.762	92
		Deep sand/seagrass	-0.511	3.067	93
V13	153	Shallow coral/rock	-0.399	2.541	89
		Shallow sand/seagrass	-0.399	2.541	89
		Deep coral/rock	-0.413	2.659	102
		Deep sand/seagrass	-0.431	2.763	107
V16	158	Shallow coral/rock	-0.194	1.789	209
		Shallow sand/seagrass	-0.194	1.789	209
		Deep coral/rock	-0.332	2.400	144
		Deep sand/seagrass	-0.231	2.004	228

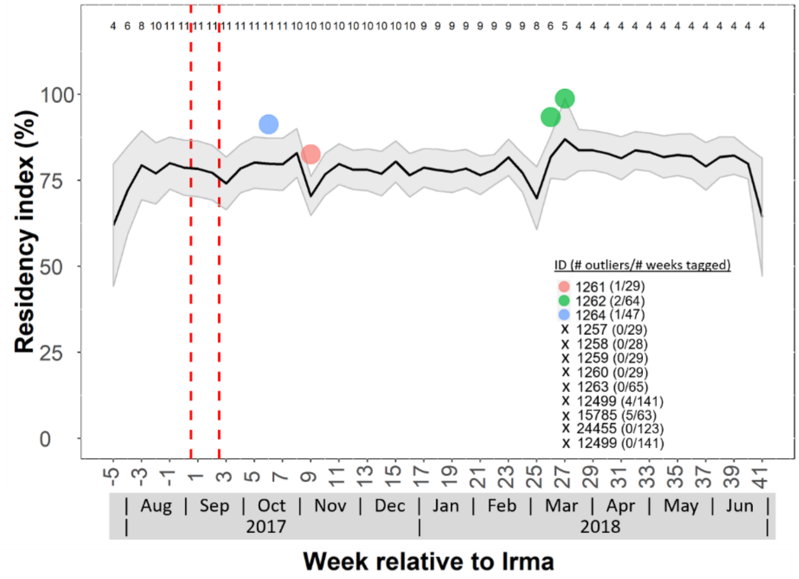

Figure Supplementary 1: Mean weekly residency indices of juvenile hawksbill turtles. The red dotted vertical lines correspond to Hurricane Irma (left) and Maria (right). The grey shaded area represents the standard error of the mean. Weekly sample sizes (i.e., number of individuals) are indicated at top of plot. Only a subset of data (i.e., week -5 to 41 relative to Hurricane Irma) is plotted for clarity. Outliers are plotted for each individual unless they occurred outside the period above, in which case they are summarized within the legend and indicated with an x.

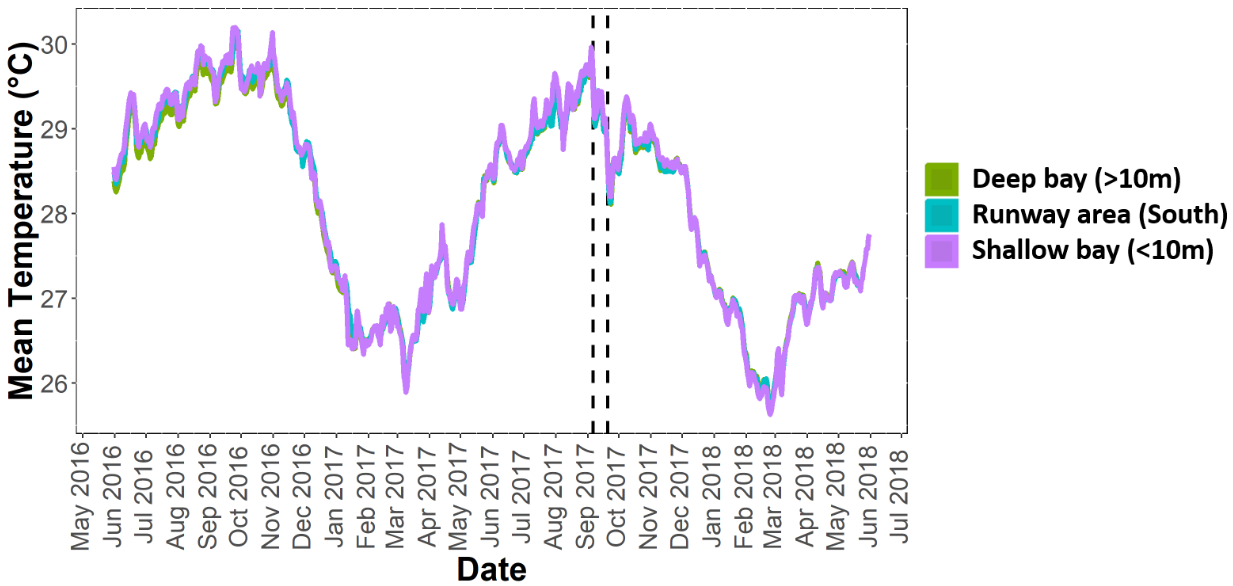

Figure Supplementary 2: Mean daily water temperature (n = 12 loggers) in the study site (Brewers Bay) between 2016-2018. The vertical dashed lines correspond to Hurricanes Irma (Sep 6, 12:00 local time) and Maria (Sep 20, 02:00 local time), respectively.

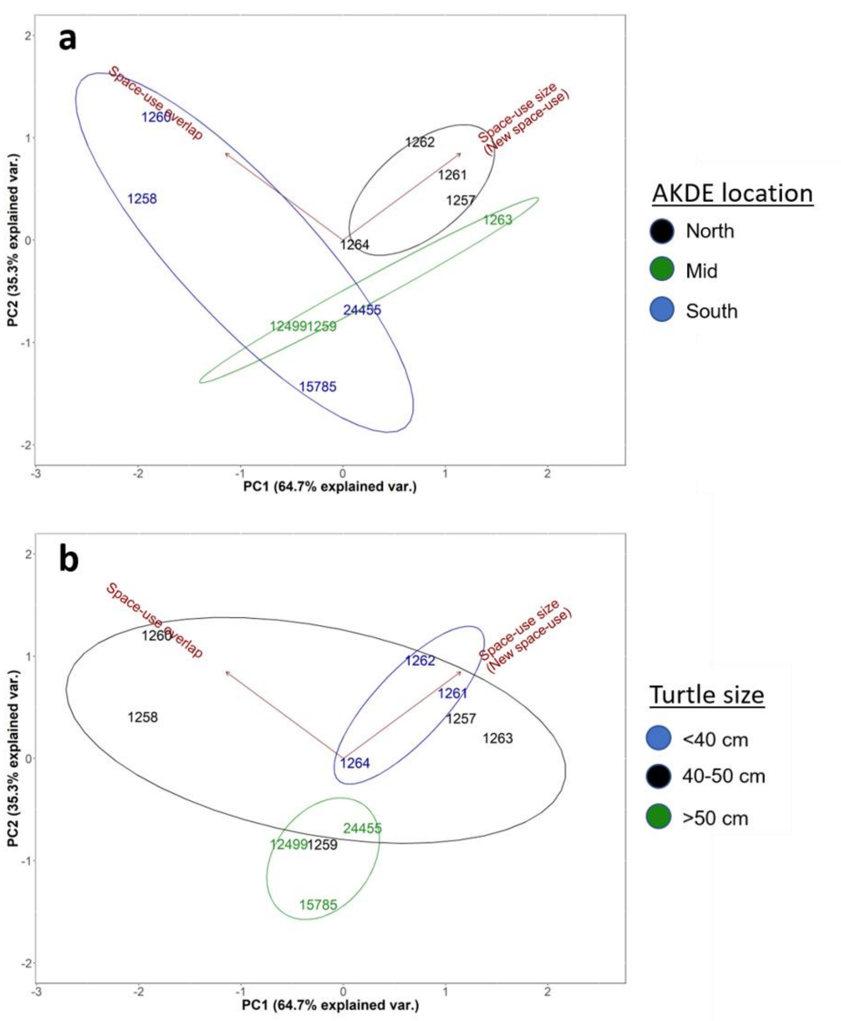

Figure Supplementary 3: Principal component analysis (PCA) of weekly ranked space-use metrics (i.e., space-use size, space-use overlap, and new space-use size) relative to the week after Hurricane Irma for juvenile hawksbill turtles. Only 95% AKDEs were included because they were strongly correlated with equivalent 50% AKDE metrics. Similarly, new space-use size was positively correlated to overall weekly space-use size. The PCA is presented twice with main location of occurrences grouped (i.e., North, South, and mid-portion of Brewers Bay; a) and size-classes grouped (i.e., <40 cm, 40-50 cm, and >50 cm carapace length; b).

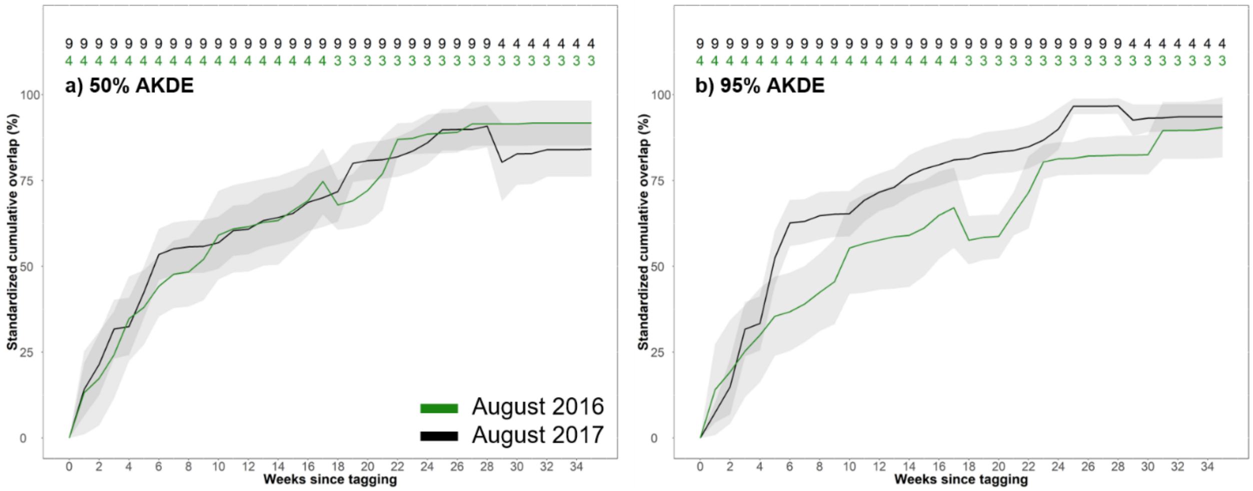

Figure Supplementary 4: Mean 50% (a) and 95% (b) cumulative AKDEs for juvenile hawksbill turtles tagged in August 2016 and July/August 2017. Weekly sample sizes (i.e., number of individuals) are indicated at top of plot.

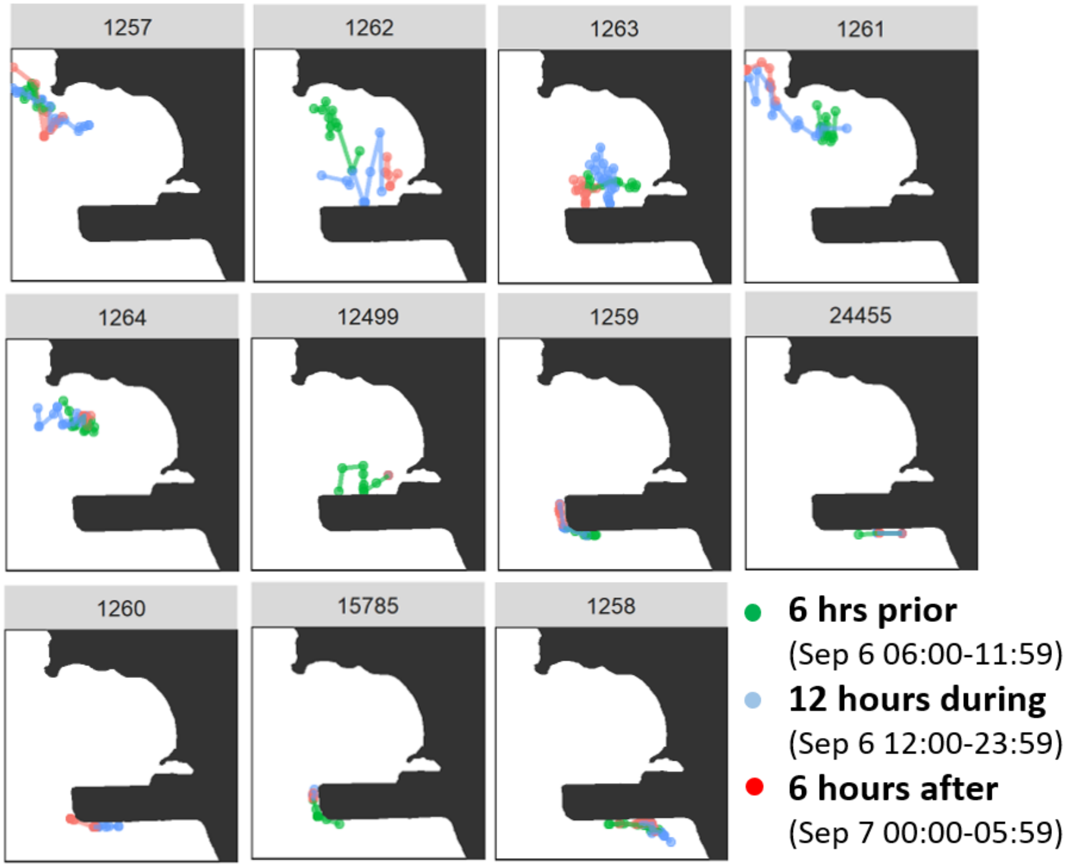

Figure Supplementary 5: Movement tracks between 30-min COAs for each juvenile hawksbill turtle at short-term intervals before, during, and after Hurricane Irma.

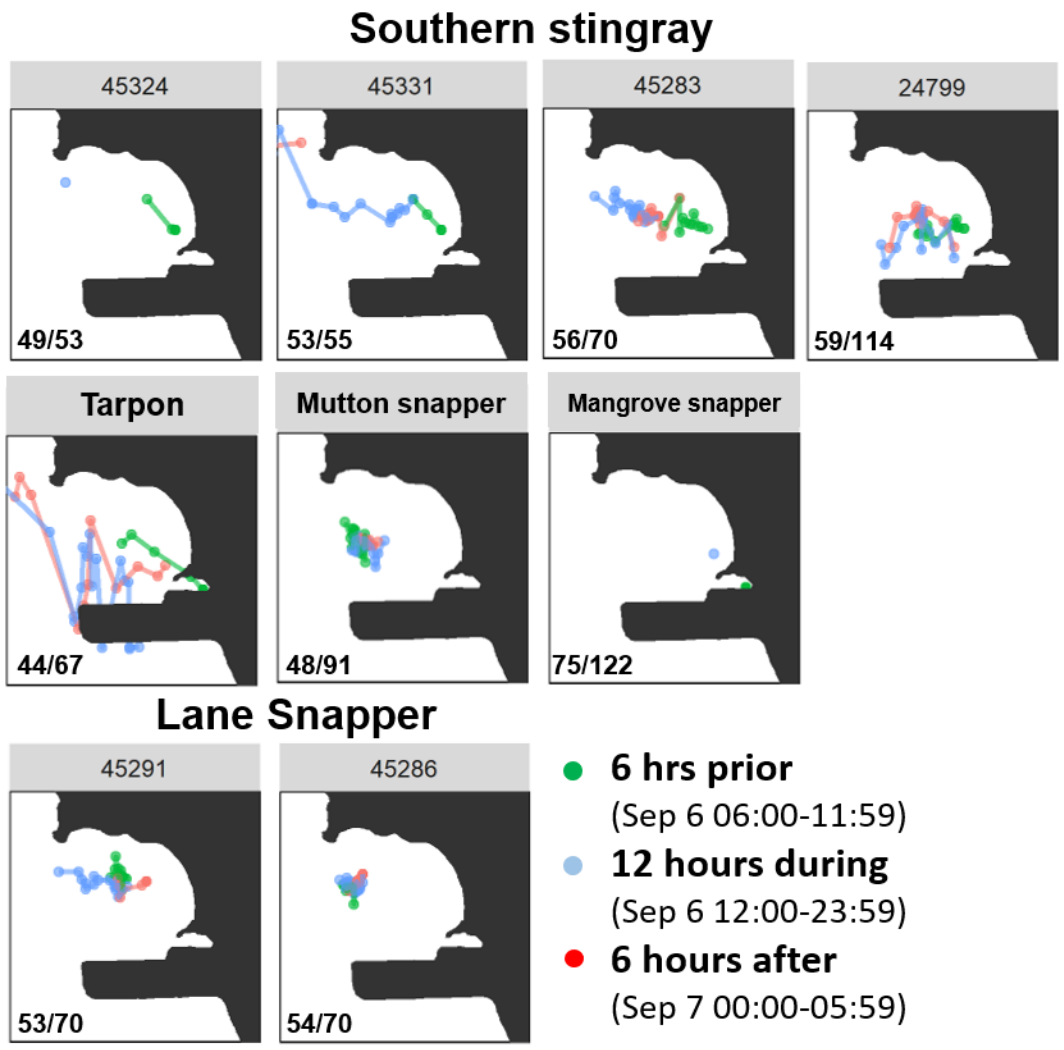

Figure Supplementary 6: Movement tracks between 30-min COAs for each fish and ray at short-term intervals before, during, and after Hurricane Irma. Each individual is identified at the top of each sub-plot; the numbers at the bottom represent the week of Hurricane Irma since tagging relative to the number of weeks the individual was tracked.

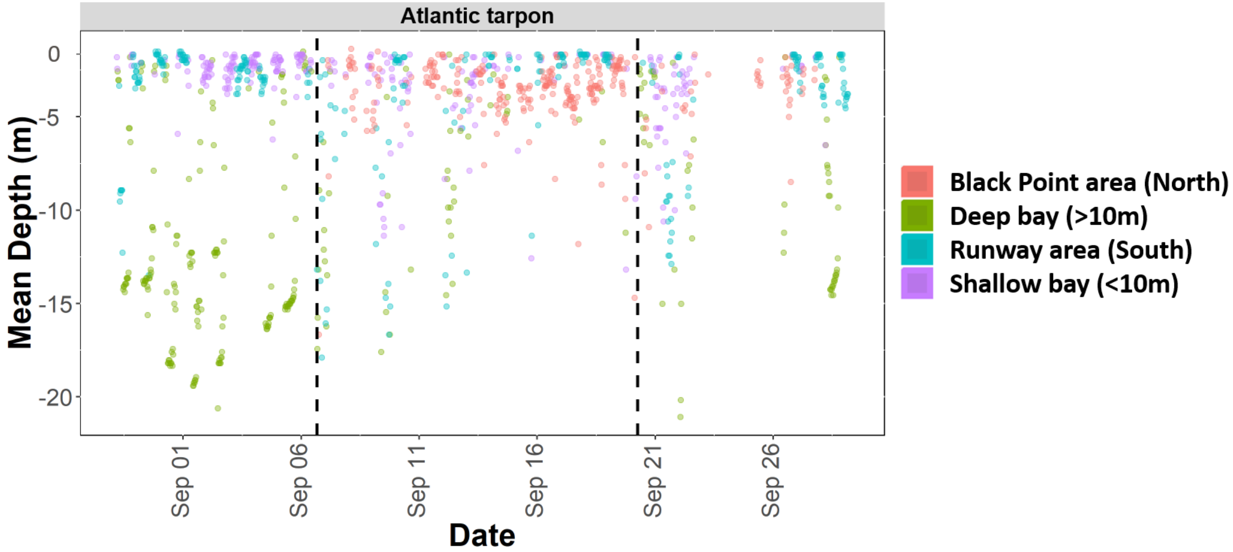

Figure Supplementary 7: Mean 30-min centres of activity (COAs) depth-use for the tarpon during September 2017. Each coloured point corresponds to a specific area within Brewers Bay. The vertical dashed lines correspond to Hurricanes Irma (a: Sep 6, 12:00 local time; b: Sep 6) and Maria (a: Sep 20, 02:00 local time; b: Sep 20), respectively.

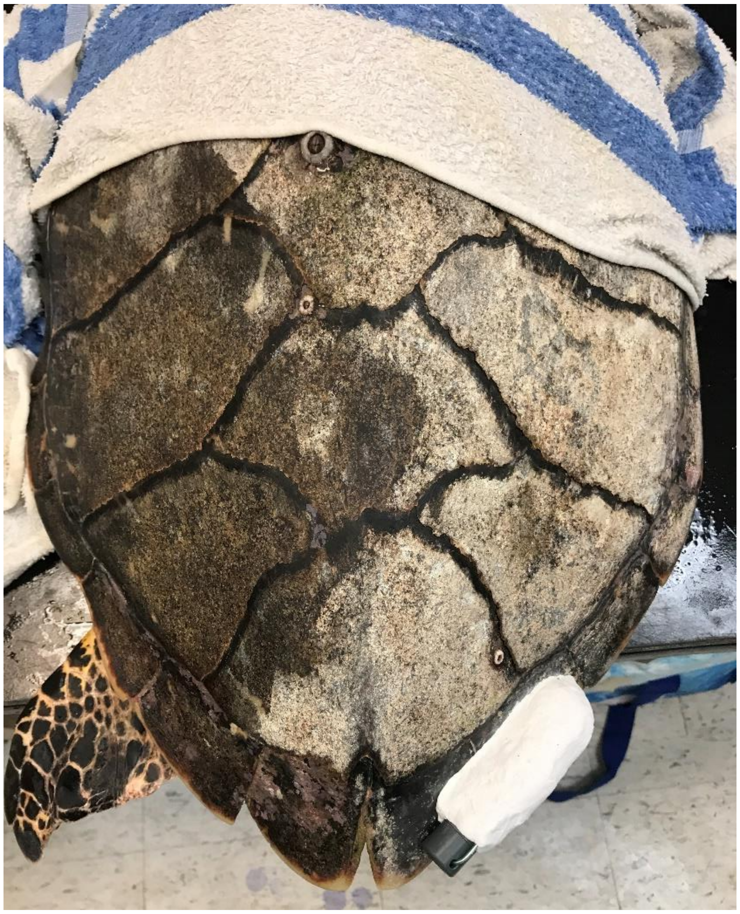

Figure Supplementary 8: Attachment method for acoustic transmitters. Transmitter was attached to two marginal scutes (adjacent to the postcentral scutes) via plastic coated wire after drilling two small holes at the extremity of one of the scutes (visible at the bottom left of the transmitter in photo) and marine putty (white material).
